# Nuclear Medicine in Times of COVID-19: How Radiopharmaceuticals Could Help to Fight the Current and Future Pandemics

**DOI:** 10.3390/pharmaceutics12121247

**Published:** 2020-12-21

**Authors:** Felix Neumaier, Boris D. Zlatopolskiy, Bernd Neumaier

**Affiliations:** 1 Forschungszentrum Jülich GmbH, Institute of Neuroscience and Medicine, Nuclear Chemistry (INM-5), Wilhelm-Johnen-Str., 52428 Jülich, Germany; boris.zlatopolskiy@uk-koeln.de (B.D.Z.); b.neumaier@fz-juelich.de (B.N.); 2 Institute of Radiochemistry and Experimental Molecular Imaging, Faculty of Medicine and University Hospital Cologne, University of Cologne, Kerpener Str. 62, 50937 Cologne, Germany; 3 Max Planck Institute for Metabolism Research, 50931 Cologne, Germany

**Keywords:** molecular imaging of SARS-CoV-2, radionuclide therapy of COVID-19, PET-based antiviral drug development, diagnostic radionuclides, therapeutic radionuclides, positron emission tomography (PET), single photon emission computer tomography (SPECT), radioimmunotherapy (RIT)

## Abstract

The emergence and global spread of COVID-19, an infectious disease caused by the novel coronavirus SARS-CoV-2, has resulted in a continuing pandemic threat to global health. Nuclear medicine techniques can be used for functional imaging of (patho)physiological processes at the cellular or molecular level and for treatment approaches based on targeted delivery of therapeutic radionuclides. Ongoing development of radiolabeling methods has significantly improved the accessibility of radiopharmaceuticals for in vivo molecular imaging or targeted radionuclide therapy, but their use for biosafety threats such as SARS-CoV-2 is restricted by the contagious nature of these agents. Here, we highlight several potential uses of nuclear medicine in the context of SARS-CoV-2 and COVID-19, many of which could also be performed in laboratories without dedicated containment measures. In addition, we provide a broad overview of experimental or repurposed SARS-CoV-2-targeting drugs and describe how radiolabeled analogs of these compounds could facilitate antiviral drug development and translation to the clinic, reduce the incidence of late-stage failures and possibly provide the basis for radionuclide-based treatment strategies. Based on the continuing threat by emerging coronaviruses and other pathogens, it is anticipated that these applications of nuclear medicine will become a more important part of future antiviral drug development and treatment.

## 1. Introduction

At the end of 2019, severe acute respiratory syndrome coronavirus 2 (SARS-CoV-2), the etiological agent of coronavirus disease 2019 (COVID-19), emerged in Wuhan, China, and quickly spread to virtually every country. The clinical presentation of COVID-19 ranges from asymptomatic infection to severe respiratory failure, septic shock and multiple organ failure, with dry cough, fever and fatigue occurring in most symptomatic cases [1]. Although first emergency use authorizations for specific vaccines have already been issued, existing knowledge regarding supportive care and adjunctive therapy is still limited. Nuclear medicine techniques are widely used for in vivo molecular imaging or targeted radionuclide therapy, but their application to biosafety level 3 or 4 (BSL3/4) threats such as SARS-CoV-2 faces several practical challenges related to the associated risk of disease spreading. Moreover, and in contrast to high-resolution chest computed tomography (CT), which remains the preferred method for monitoring and severity assessment of COVID-19 pneumonia, the limited availability and high costs of nuclear imaging techniques mean that they cannot be routinely used in patients infected with SARS-CoV-2. With regard to preclinical studies in animals infected with contagious pathogens, attempts have been made to use self-contained isolation chambers [2,3] or imaging systems installed in BSL3/4 approved laboratories [4,5] (for review see [6]), but these approaches come with several disadvantages and are unlikely to be feasible for many current radiochemistry or imaging facilities. The main aim of the present article is to highlight potential uses of nuclear medicine in the context of SARS-CoV-2/COVID-19, with a special emphasis on applications that could also be performed in laboratories without dedicated containment measures. A second aim is to give a broad overview of the structure and mechanism of action of different experimental or repurposed antiviral drugs that could provide a starting point for the development of suitable radiopharmaceuticals. To this end, Section 2 will briefly summarize current knowledge about SARS-CoV-2 structure, tropism, infection and replication and review different classes of experimental antiviral drugs. Section 3 will focus on molecular imaging using tracers labeled with diagnostic radionuclides and describe how such radiopharmaceuticals could either be applied to investigate different aspects of SARS-CoV-2 infection or to facilitate translation of SARS-CoV-2-targeting antiviral drugs to the clinic. Section 4 will address targeted radionuclide therapy based on drugs labeled with therapeutic radionuclides and discuss if and how this approach could potentially be employed for the treatment of COVID-19.

## 2. SARS-CoV-2 Structure, Infection, Replication and Treatment

Based on genomic analysis, SARS-CoV-2 belongs to the same spade of β-coronaviruses that caused earlier outbreaks of deadly pneumonia in humans, including severe acute respiratory syndrome coronavirus (SARS-CoV) in 2002/2003 and Middle East respiratory syndrome coronavirus (MERS-CoV) in 2012 [7]. Thanks to a high degree of conservation between these viruses and intense global research efforts, the structure of SARS-CoV-2 and the most important processes underlying host cell entry and subsequent viral replication are relatively well established, and a number of experimental drugs targeting them have already been identified, as will be described in the following sub-sections.

### 2.1. Virion Structure

**Figure 1:**
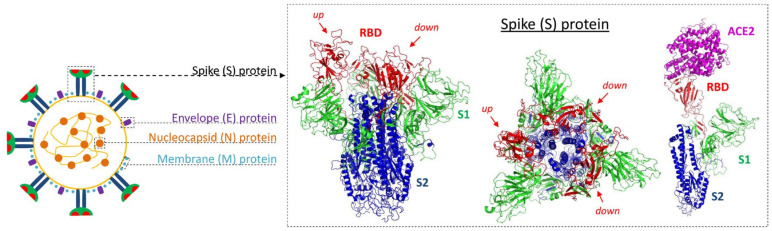
Structure of severe acute respiratory syndrome coronavirus 2 (SARS-CoV-2). Schematic representation illustrating the 4 structural proteins of SARS-CoV-2. Inset shows side (left) and top (middle) view of homotrimeric spike glycoprotein with two of the receptor binding domains (RBD, red) in their down position and one in the up position. The rightmost structure shows a single monomer with the RBD in its up position and bound to human ACE2. Protein structures visualized with PyMol using models from https://swissmodel.expasy.org.

β-Coronaviruses such as SARS-CoV-2 consist of a positive-sense, single-stranded RNA genome encapsulated by a membrane envelope and four structural proteins, which comprise the nucleocapsid (N) protein, envelope (E) protein, membrane (M) protein and spike (S) glycoprotein (Figure 1). The N protein is responsible for packaging of the viral RNA into a ribonucleoprotein complex, the so-called (nucleo)capsid, while M and E protein make up the viral envelope, from which the S proteins protrude like the spikes of a crown [8,9]. On mature viruses, the S proteins are present as homotrimers made up of three receptor-binding S1 heads on top of a trimeric membrane fusion S2 stalk [10]. Each S1 head contains a receptor-binding domain (RBD) that constantly switches between a lying-down position for immune evasion and a standing-up position for receptor recognition and binding (Figure 1 inset).

### 2.2. Cell Entry and Tissue Tropism

In their standing-up position, the S protein RBDs can bind to a target receptor on the host cell surface, which facilitates viral attachment and subsequent cell entry. The latter requires S protein priming by host cell surface proteases, a process that involves proteolytic cleavage at the S1/S2 boundary followed by S2-driven fusion of viral and cellular membrane [10]. Similar to SARS-CoV, SARS-CoV-2 has been shown to employ angiotensin-converting enzyme 2 (ACE2) as the entry receptor (Figure 1 inset) and transmembrane serine protease 2 (TMPRSS2) for S protein priming [10,11]. In humans, co-expression of ACE2 and TMPRSS2 is most prominent in epithelial cells of the upper airway and the nasal region [12,13,14,15], which is consistent with the major role of the respiratory system for SARS-CoV-2 infection and transmission. However, ACE2 is also abundantly expressed in epithelial and smooth muscle cells of virtually all organs [16], and there is evidence for the existence of additional entry receptors such as neuropilin-1, which may facilitate viral entry into the CNS [17]. Likewise, other proteases such as cathepsin B/L may be able to substitute for TMPRSS2 [11], suggesting that SARS-CoV-2 could spread via the bloodstream once it reaches circulation. In support of this assumption, endothelial cell involvement across the vascular beds of different organs has been demonstrated in a number of patients [18] and COVID-19 is increasingly recognized as a multisystem illness that can be associated with pulmonary, coagulation, cardiac, hepatic, gastrointestinal and neurological manifestations [19]. On the other hand, damage to multiple organ systems could also result from hyperinflammatory reactions or other factors [20] and direct evidence for the actual presence of the virus outside of the respiratory system remains sparse.

### 2.3. Replication in Infected Cells

**Figure 2:**
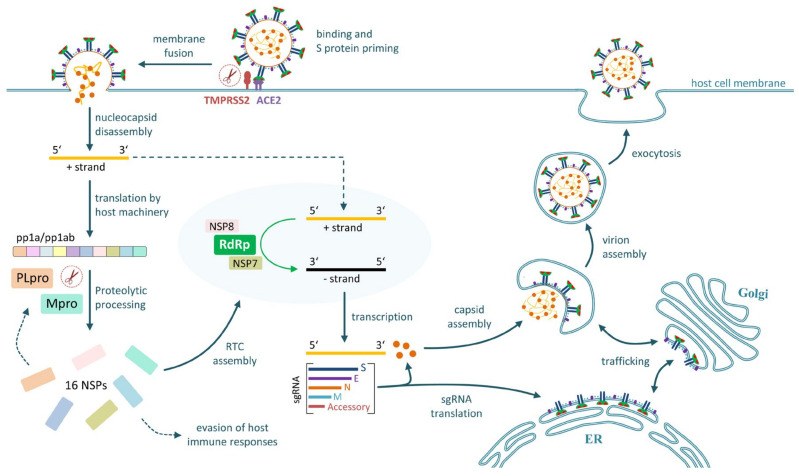
SARS-CoV-2 replication and transcription cycle in infected cells. Schematic representation of the processes involved in the replication of SARS-CoV-2 in infected host cells. For details see Section 2.2 and Section 2.3.

In tissues that express the necessary entry receptors and proteases, membrane fusion is followed by release of the nucleocapsid into the cytoplasm, where it disassembles and exposes a ~30 kb RNA strand that encodes the 4 structural proteins, 16 non-structural proteins (NSPs) and 9 accessory proteins (Figure 2). Gene 1, which occupies approximately two-third of the viral genome, is translated by host-cell ribosomes into two overlapping replicase polyproteins (pp1a and pp1ab), which are cleaved into the 16 NSPs by two virally encoded cysteine proteases [21,22]. Viral 3-chymotrypsin-like protease (3CLpro or Mpro, NSP5) is the main protease and responsible for processing 11 restriction sites (including its autolytic cleavage site) on the pp1ab protein [21,22,23]. The remaining three restriction sites are cleaved by viral papain-like protease (PLpro, NSP3), which is also involved in the evasion of host antiviral immune responses [21,24]. Replication is initiated by the assembly of a replication/transcription complex (RTC), which concentrates the necessary NSPs in a microenvironment close to the viral RNA [25]. The central component of the RTC, formed by viral RNA-dependent RNA-polymerase (RdRp, NSP12) and its cofactors NSP7 and NSP8, has the capacity to generate full-length negative-strand RNA chains that serve as templates for the synthesis of positive-strand genomic (gRNA) and subgenomic (sgRNA) RNA [21,26] (Figure 2). Apart from accessory proteins, the sgRNA encodes the four structural proteins, which are translated and released into the cytosol, where they either insert into the endoplasmic reticulum (M, E and S proteins) or assemble nascent gRNA into nucleocapsid complexes (N protein) (Figure 2). Budding and progeny virion assembly occur after trafficking of M, E and S proteins to the Golgi apparatus, where they envelope the nucleocapsid complexes to form new virions, which are released by exocytosis (Figure 2) and can infect additional host cells.

### 2.4. Experimental Drugs Targeting SARS-CoV-2 Cell Entry

**Figure 3:**
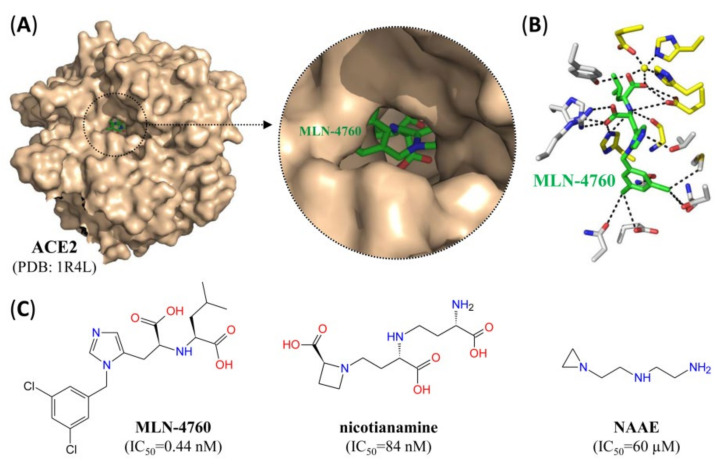
Mechanism of action and structure of small molecule ACE2 inhibitors. (**A**) Surface representation of the crystal structure of ACE2 with the small molecule inhibitor MLN-4760 (green) bound to the active site. Inset: close-up view showing part of the inhibitor bound to the active site. (**B**) Stick representation of MLN-4760 (green) bound to residues at the active site. Catalytic residues and the active site zinc ion are indicated in yellow. Dashed black lines indicate formation of hydrogen bonds. (**C**) Structure of different small molecule inhibitors of ACE2 and IC_50_ values for suppression of human ACE2.

Apart from vaccination, most prophylactic approaches against SARS-CoV-2 infection have either focused on disrupting the interaction between S protein and the entry receptor ACE2 or on suppressing host cell surface proteases required for S protein priming. With regard to the former, a number of RBD-specific neutralizing antibodies obtained from recovered COVID-19 patients or immunized transgenic animals have been shown to effectively inhibit virus entry in vitro with picomolar to sub-micromolar IC_50_ values [27,28,29] (Table 1). In addition, the small molecule anti-influenza drug arbidol binds to a region on the S2 domain of the S protein, thereby preventing trimerization and suppressing SARS-CoV-2 cell entry in vitro [30]. Finally, several small molecules [31,32,33] (Figure 3) and the peptide DX-600 (IC_50_ = 10.1 µM) [34,35,36] have been shown to inhibit ACE2, and at least some of them also suppressed cellular entry of SARS-CoV pseudotyped viruses in vitro [33]. Given the important physiological roles of ACE2, treatment with these inhibitors does not seem to be a viable therapeutic modality, but they could be useful for certain imaging applications (see Section 3.2). Consistent with a dependence of membrane fusion on S protein priming by host cell surface proteases, suppression of TMPRSS2 with the serine protease inhibitor camostat has also been shown to reduce cellular entry of SARS-CoV and SARS-CoV-2 in vitro [11,39]. However, complete prevention of cell entry was only observed after combined suppression of TMPRSS2 and cathepsin, supporting the notion that S protein priming can be mediated by multiple proteases [11,39].

**Figure 4:**
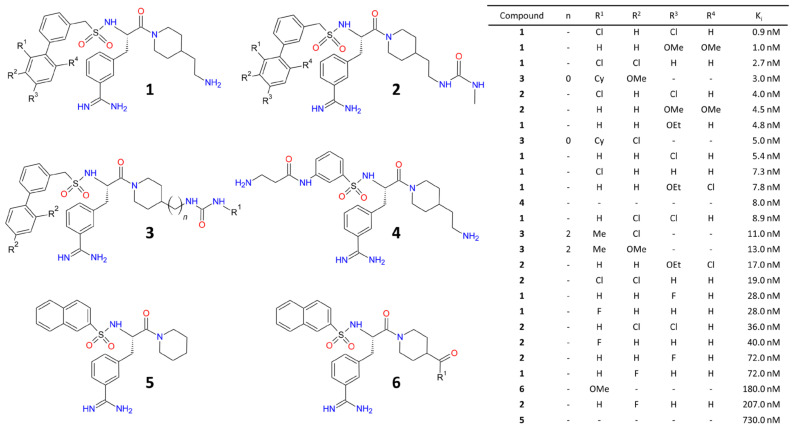
Structure–activity relationship of small molecule TMPRSS2 inhibitors. Shown are various synthetic inhibitors with sulfonylated 3-amindinophenylalanylamide moieties. For details and additional structures, see [42].

An overview of several experimental small molecule inhibitors of TMPRSS2 and their structure-activity relationship is provided in Figure 4. A comprehensive review of the many available cathepsin B and L inhibitors and their mechanisms of action is beyond the scope of the present article but can be found elsewhere [40,41].

### 2.5. Experimental Drugs Targeting SARS-CoV-2 Replication

**Figure 5:**
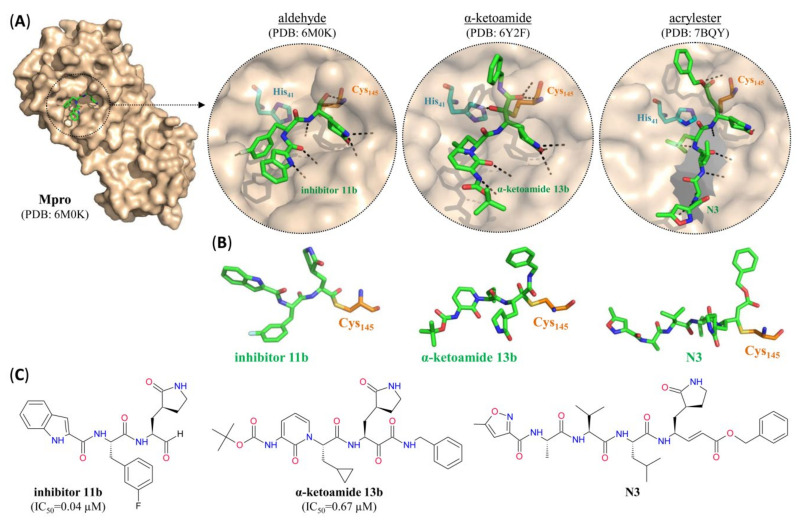
SARS-CoV-2 Mpro peptidomimetic inhibitors with different electrophilic warheads. (**A**) Surface representation of one protomer from the crystal structure of SARS-CoV-2 Mpro with the peptidomimetic inhibitor 11b (green) bound to the active site. Insets: close-up view of the active site after covalent modification by (from left to right) inhibitor 11b with an aldehyde warhead, α-ketoamide 13b with an α-ketoamide warhead or N3 with an acrylate warhead (green). Residues forming the catalytic dyad are indicated in turquoise (His_41_) or orange (Cys_145_) and hydrogen bonds are indicated by dashed black lines. (**B**) Stick representations of (from left to right) aldehyde inhibitor 11b, α-ketoamide 13b and acrylate N3 (green) covalently bound to the catalytic cysteine residue (orange). (**C**) Structure of (from left to right) aldehyde inhibitor 11b, α-ketoamide 13b and acrylate N3. For pharmacological properties see Table 2. For the structures of additional petidomimetic inhibitors with aldehyde or α-ketoamide warheads listed in Table 2, see Appendix A at the end of the article.

Several of the NSPs involved in SARS-CoV-2 replication are expected to be excellent targets for the treatment of COVID-19 in infected patients. In particular, a number of novel or repurposed drugs have been identified that bind with high-affinity to one or, in some cases, both of the virus-specific cysteine proteases, thereby preventing cleavage of the replicase polyproteins and assembly of the RTC. They can be broadly classified into peptide analogues that mimic part of the peptide substrate of the proteases and various small molecule drugs. Essentially all of the peptidomimetics targeting SARS-CoV-2 Mpro are covalent inhibitors with electrophilic aldehyde [22,43], α-ketoamide [43,44] or acrylate [23,45] warheads, which trap the active site cysteine residue (Cys_145_) of the protease (Figure 5). Although many of them exhibit excellent binding affinity and antiviral activity with little cytotoxicity in in vitro assays (Table 2), their pharmacokinetic (PK) and biodistribution (absorption, distribution, metabolism and excretion or ADME) properties as well as their selectivity with regard to host-expressed proteins remain to be established.

**Figure 6:**
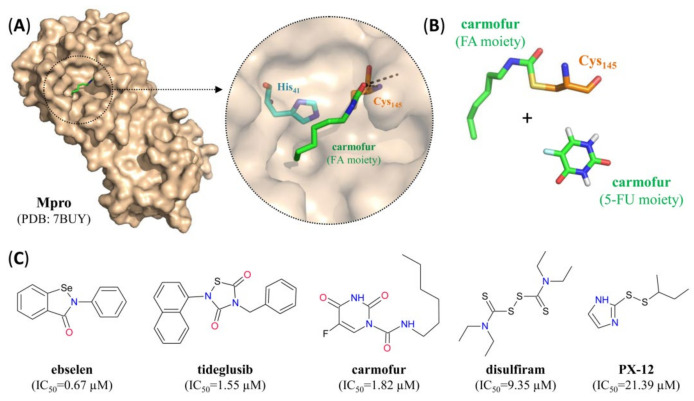
SARS-CoV-2 Mpro small molecule covalent inhibitors. (**A**) Surface representation of one protomer from the crystal structure of SARS-CoV-2 Mpro with the carmofur fatty acid moiety (green) bound to the active site. Inset: close-up view of the inhibitor-bound active site, with residues forming the catalytic dyad indicated in turquoise (His_41_) or orange (Cys_145_) and hydrogen bonds indicated by dashed black lines. (**B**) Stick representation of the fatty acid (FA) moiety of carmofur (green) covalently bound to the catalytic cysteine residue (orange). In addition, the 5-fluorouracil (5-FU) moiety released during binding of carmofur to the active site is also shown. (**C**) Structure of different small molecule covalent SARS-CoV-2 Mpro inhibitors. For pharmacological properties, see Table 2.

The antineoplastic agent carmofur [23,46] and a number of sulfur- or selenium-containing repurposed drugs such as ebselen and disulfiram [23] have also been shown or are thought to act by covalent modification of Cys_145_ (Figure 6). Members of this group are often promiscuous binders and may act by more than a single mechanism of action, as exemplified by two recent preprints on ebselen and disulfiram [47,48], which indicate that both compounds can also suppress PLpro through a combination of covalent modification and ejection of critical Zn^2+^ ions (Table 3).

**Figure 7:**
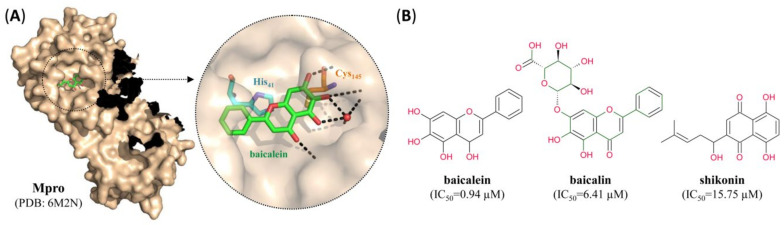
SARS-CoV-2 Mpro small molecule non-covalent inhibitors. (**A**) Surface representation of one protomer from the crystal structure of SARS-CoV-2 Mpro with baicalein (green) bound to the active site. Inset: close-up view of the inhibitor-bound active site, with residues forming the catalytic dyad indicated in turquoise (His_41_) or orange (Cys_145_) and hydrogen bonds indicated by dashed black lines. (**B**) Structure of different small molecule non-covalent SARS-CoV-2 Mpro inhibitors. For pharmacological properties, see Table 2.

Most of the known non-covalent inhibitors of Mpro are plant-derived products such as baicalein, baicalin [49] or shikonin [23], which bind to residues near the active site and prevent access of the replicase polyproteins to Cys_145_ (Figure 7). However, several structurally related flavonoids which have previously been shown to be potent non-covalent inhibitors of Mpro from SARS-CoV [50] and MERS-CoV [51] might also be effective against Mpro from SARS-CoV-2.

**Figure 8:**
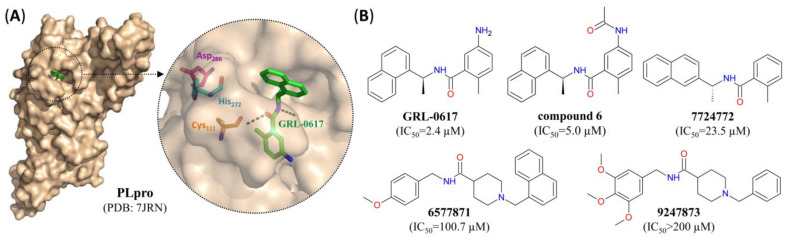
SARS-CoV-2 PLpro small molecule non-covalent inhibitors. (**A**) Surface representation of the crystal structure of PLpro from SARS-CoV-2 with GRL-0617 (green) bound to the active site. Inset: close-up view of the inhibitor-bound active site, with residues forming the catalytic triad indicated in turquoise (His_272_), orange (Cys_111_) or purple (Asp_286_) and hydrogen bonds indicated by dashed black lines. (**B**) Structure of different small molecule non-covalent SARS-CoV-2 PLpro inhibitors. For pharmacological properties, see Table 3.

A similar mechanism of action is involved in the suppression of SARS-CoV-2 PLpro by various napthylamines [24,52], which also bind non-covalently to residues near the active site and act as a shield between substrate and the catalytic triad of the protease (Figure 8).

**Figure 9:**
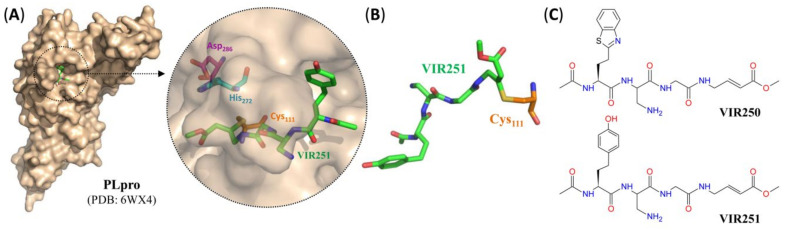
SARS-CoV-2 PLpro peptidomimetic inhibitors with an electrophilic vinyl methyl ester warhead. (**A**) Surface representation of the crystal structure of PLpro from SARS-CoV-2 with VIR251 (green) bound to the active site. Inset: close-up view of the inhibitor-bound active site, with residues forming the catalytic triad indicated in turquoise (His_272_), orange (Cys_111_) or purple (Asp_286_) and hydrogen bonds indicated by dashed black lines. (**B**) Stick representation of VIR251 (green) covalently bound to the catalytic cysteine residue (orange). (**C**) Structure of different peptidomimetic SARS-CoV-2 PLpro inhibitors with electrophilic vinyl methyl ester warhead. For pharmacological properties, see Table 3.

In addition, in a recent preprint [53] for which crystal structures were already deposited, two peptidomimetic vinyl methyl esters were shown to suppress SARS-CoV-2 PLpro by covalent modification of the catalytic cysteine residue (Cys_111_) of the protease (Figure 9).

Another important target for SARS-CoV-2 treatment is the viral polymerase RdRp, which is suppressed by broad-spectrum antiviral drugs such as remdesivir. The main metabolite of the latter is bioactivated by intracellular phosphorylation and incorporated into the viral RNA, resulting in RNA synthesis arrest by delayed chain termination [54].

Finally, certain metallodrugs such as the commonly used antimicrobial agent ranitidine bismuth citrate have been shown to suppress SARS-CoV-2 replication in vitro and in vivo through an irreversible, Bi^3+^-induced displacement of Zn^2+^ ions from viral helicase (NSP13) and possibly other Zn^2+^-containing SARS-CoV-2 NSPs [55,56].

## 3. Application of Nuclear Imaging in the Context of SARS-CoV-2

**Figure 10:**
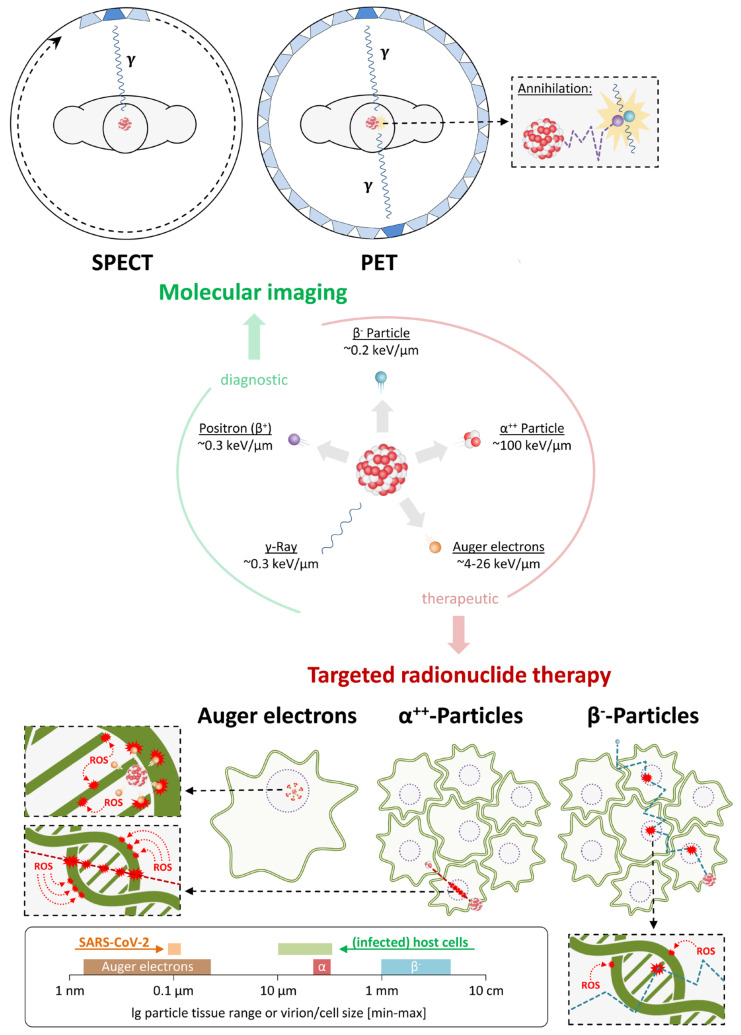
Overview of diagnostic and therapeutic nuclear medicine techniques. Shown are different types of radiation and their approximate linear energy transfer (**middle**) as well as their use for molecular imaging by PET and SPECT (**top**) or for targeted radionuclide therapy (**bottom**). Inset in the bottom left compares the tissue range of particles emitted by different types of therapeutic radionuclides with the size of SARS-CoV-2 virions and (infected) host cells.

Nuclear imaging techniques such as single-photon emission computer tomography (SPECT) and positron emission tomography (PET) are based on the use of tracers labeled with diagnostic radionuclides (Table 4), which can be detected non-invasively by measuring γ-rays emitted during (or shortly after) their decay. Radionuclides used for SPECT imaging decay under direct emission of γ-rays (^99m^Tc) or by electron capture with subsequent emission of γ-rays (^111^In, ^123^I), which are detected by gamma cameras that rotate around the subject [57,58,59] (Figure 10 top left). PET imaging, on the other hand, is performed with a ring of detectors surrounding the subject and radionuclides that emit positrons (β^+^-particles), which travel a short distance in tissues before they collide with an electron and undergo annihilation (Figure 10 top right) [57,58,59,60]. The latter produces a pair of 511 keV γ-rays that are released at almost 180 degrees to each other. Since they strike opposite detectors of the PET scanner, the annihilation event can be localized to a point somewhere along the line of response joining the two detectors [57,58,60]. Following a number of pre-processing steps, the data from a large number of events registered during a SPECT or PET scan can be used to reconstruct a cross-sectional image of tracer distribution. Since essentially all drugs and biomolecules can, in principle, be labeled with PET or SPECT radionuclides, nuclear imaging techniques allow for functional imaging of various physiological processes at the cellular or molecular level and have become an integral part of preclinical research and clinical decision making [60,61,62].

A particular advantage of these techniques is that only tracer quantities of a radiolabeled drug need to be administered for PK and ADME studies, so that they can be performed early (i.e., before phase I trials) in patients [63,64]. For a more detailed description of nuclear imaging techniques and their many applications, the reader is referred to several previous reviews on the topic [58,60,65,66,67]. Here, we will focus on how PET or SPECT imaging could potentially be used to visualize host responses to SARS-CoV-2 infection (Section 3.1.), to localize host molecules involved in viral infection (Section 3.2.), to facilitate antiviral drug development and translation to the clinic (Section 3.3.) or to detect SARS-CoV-2-infected tissues and/or the free virus (Section 3.4).

### 3.1. Imaging of Host Responses to SARS-CoV-2 Infection

At present, the most widely used PET tracer is 2-[^18^F]fluoro-2-deoxyglucose ([^18^F]FDG), a glucose derivative that is taken up by cells and phosphorylated but not further metabolized, so that it accumulates in cells with high rates of glycolysis. As such, [^18^F]FDG can be used to visualize cells or tissues with increased glucose metabolism, such as active neurons [68,69] and many tumors [70]. Foci of apparently increased glycolytic activity (due to nonspecific tracer uptake by white blood cells) are also characteristic for infectious and inflammatory processes [71,72,73], and [^18^F]FDG PET has been shown to detect lung abnormalities in asymptomatic patients [73,74,75](reviewed in [76]) and non-human primates [5] infected with SARS-CoV-2. In previous studies, [^18^F]FDG PET imaging has also been used to identify inflammatory patterns of monkeypox virus in non-human primates [77,78]. Moreover, even though [^18^F]FDG PET cannot reliably distinguish between infection and other inflammatory processes, increased glucose metabolism in the lungs of ferrets infected with the pandemic influenza virus H1N1 has been correlated with viral titers [79]. More specific PET tracers that can be used to detect inflammation include [^18^F]AzaFol and R-[^11^C]PK11195, which bind to receptors expressed on (activated) macrophages [80,81]. The latter has previously been employed to study viral encephalitis in non-human primates infected with simian immunodeficiency virus (SIV) [82]. Likewise, radiolabeled polyclonal immunoglobulins, which are extravasated and retained at sites of inflammation [83], have been used for imaging of inflammation in patients infected with HIV [84]. Another approach that involves direct (ex vivo) or indirect (in vivo) radiolabeling of leukocytes has been shown to provide up to 90% sensitivity for the detection of certain acute and chronic infections [85,86]. In a recent case report, [^99m^Tc]Tc-leukocyte scintigraphy in a patient with spiking fevers revealed increased uptake in the right upper thorax and both lungs, which was later confirmed to reflect pulmonary infection with SARS-CoV-2 [87]. While the routine application of these techniques for monitoring disease progression in patients suffering from COVID-19 is evidently restricted by the high costs and limited availability of PET scanners, animal or human studies with these probes could facilitate a more throughout understanding of tissue and organ responses to SARS-CoV-2 infection and help to delineate the role of host responses for the progression and outcome of acute SARS-CoV-2 infection. Given that there is increasing evidence for long-term impairments in a considerable proportion of recovered COVID-19 patients [20,88,89,90], monitoring of inflammatory markers or tissue responses to inflammation by PET and SPECT could also help to determine if and how chronic inflammatory processes contribute to these phenomena.

### 3.2. Imaging of Host Molecules Involved in SARS-CoV-2 Infection

Although the expression of SARS-CoV-2-targeted host cell surface proteins has been the subject of many studies [12,13,14,15,16], the exact cells and tissues at risk of infection are still a matter of debate. In addition, it is still unclear whether differences in the expression of entry receptors such as ACE2 due to e.g., treatment with ACE2-increasing drugs, are responsible for differences in the sensitivity to COVID-19 between different patient populations [91,92]. Radiolabeled analogs of the compounds shown in Figure 3 and Figure 4 or described elsewhere [40,41,42] could be used to study the distribution of SARS-CoV-2 entry receptors and/or the proteases required for S protein priming in different tissues and/or patient populations. Even more importantly, PET or SPECT imaging with radiolabeled SARS-CoV-2 S protein could provide an in vivo read-out of tissues that express any of the entry receptors, even without knowledge of their molecular counterparts. Another related application of radiolabeled S protein are autoradiographic studies on the distribution and/or identity of SARS-CoV-2 entry receptors in e.g., tissue slices. Finally, it might also be possible to combine the binding motifs of ligands targeting, e.g., ACE2 and TMPRSS2 into a dual-targeted molecular imaging agent that only binds with high affinity to cells co-expressing both proteins in close spatial proximity [93,94]. Given that inhibitors of ACE2 bound to the active site appear to be buried deep in the center of the protein (Figure 3A,B), such an approach would most likely require novel drugs targeting a region on the surface of the protein. Regardless of the exact approach used, a clear advantage of imaging studies on SARS-CoV-2-targeted host proteins would be that they could be performed in healthy animals or human subjects, thus avoiding the need for special containment measures.

### 3.3. PET- or SPECT-Based Antiviral Drug Development

**Figure 11:**
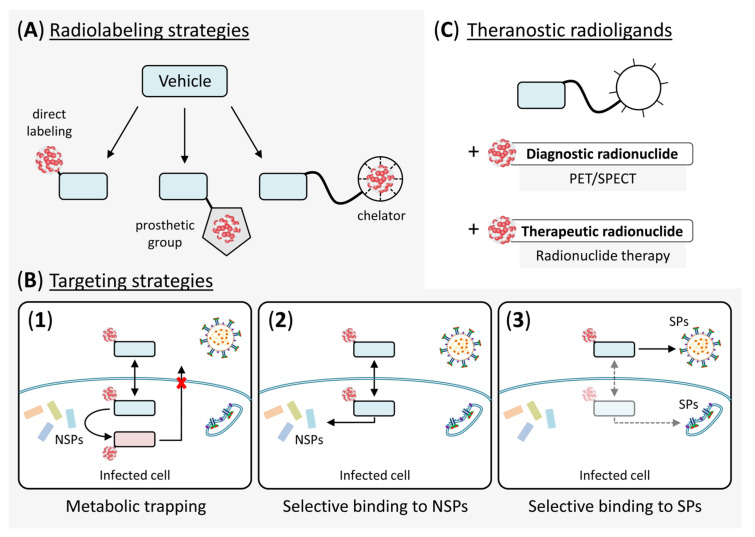
Radiolabeling and targeting strategies for virus-specific imaging and/or radionuclide therapy. (**A**) Coupling of radionuclides to a suitable vehicle for delivery to the target structures can be achieved through direct radiolabeling of the vehicle (left), by conjugation of the vehicle with radiolabeled prosthetic groups (middle) or by conjugation of the vehicle with a chelator for radiometal complexation (right). (**B**) Delivery of radionuclides to virus-infected cells and/or the free virus could be achieved with (**1**) vehicles that are metabolized by virus-encoded non-structural proteins (NSPs) and selectively retained in infected cells, (**2**) vehicles that selectively bind to virus-encoded NSPs in infected cells or (**3**) vehicles that selectively bind to virus-encoded structural proteins (SPs) on the free virus and (for vehicles that can cross the cell membrane) in infected cells. (**C**) Theranostic radioligands are usually based on conjugation of a vehicle with a chelator for complexation of either diagnostic or therapeutic radiometals, allowing for application of the same conjugates for imaging and radionuclide therapy.

High-throughput screening and computational techniques have improved the drug discovery process, but preclinical and clinical studies remain a significant obstacle for rapid translation of candidate drugs to the clinic. This can be a particular problem for the introduction of antiviral drugs, since many classical methods of drug development cannot be applied to BSL3/4 pathogens such as SARS-CoV-2. Since nuclear imaging techniques can be used to track the fate of radiolabeled drugs in vivo, PET and SPECT could evidently help to reduce the incidence of late-stage failures and to overcome several of the potential bottlenecks that might hamper translation of candidate antiviral drugs to the clinic. For example, as already noted in Section 2.5, the in vivo properties of most experimental SARS-CoV-2-targeting drugs as well as their affinity towards host proteins are currently unknown. PET or SPECT imaging with radiolabeled analogs of these drugs in healthy animals and possible humans could be used to determine their pharmacokinetic properties, verify adequate delivery to the intended target tissues and visualize potential interactions with host molecules, thereby accelerating the identification of optimal lead structures. A PET- or SPECT-based approach that involves imaging in healthy subjects, complemented by computational methods and suitable in vitro assays (see next section), could also streamline the subsequent lead optimization process and help to identify optimal routes of administration or dosing schedules, thereby accelerating the conversion of candidate drugs into molecules with in vivo activity. The feasibility of such approaches is vividly illustrated by a number of previous PET- or SPECT-based pharmacokinetic and biodistribution studies with radiolabeled anti-retroviral or anti-influenza drugs in healthy animals [61,95,96] or human subjects [62,97]. Moreover, most or all of the compounds described in Section 2 could either be directly radiolabeled, conjugated with radiolabeled prosthetic groups or coupled to a suitable chelator for radiometal chelation (Figure 11A). Given that the mechanisms of action of most compounds have been established and high resolution crystal structures of the ligand-bound targets are usually available, it should also be relatively easy to identify optimal positions for radiolabeling and to develop probes that retain high-affinity binding to the target proteins. For example, the peptidomimetic aldehyde inhibitors 11a and 11b (Figure 5 and Appendix A) could probably be labeled using a prosthetic group or chelator coupled to the indole ring, while several of the α-ketoamides (Figure 5 and Appendix A) as well as the acrylate N3 (Figure 5) possess a phenyl group that protrudes away from the ligand-bound protease and might serve as a site for radiolabeling. Apart from facilitating antiviral drug development, data obtained with the resulting probes should also provide a basis for the identification of SARS-CoV-2-specific radiopharmaceuticals, which could, in turn, be used to non-invasively assess antiviral drug efficiency and SARS-CoV-2 tissue tropism in infected subjects (see next section) or to deliver a therapeutic radionuclide to the virus or virus-infected cells (see Section 4).

### 3.4. SARS-CoV-2-Specific Nuclear Imaging

The use of nuclear imaging as a first-line diagnostic modality for COVID-19 is currently unfeasible, but PET and SPECT could significantly advance the understanding of SARS-CoV-2 tissue tropism, infection and treatment. There are three principle approaches that can be used for virus-specific molecular imaging, which comprise: (1) radiolabeling of molecules that are metabolized by a virus-encoded non-structural protein and selectively retained in infected cells (Figure 11B1), (2) radiolabeling of molecules that selectively bind to a virus-encoded non-structural protein (Figure 11B2) or (3) radiolabeling of molecules that selectively bind to a virus-encoded structural protein (Figure 11B3). The first approach is exemplified by PET imaging of herpes simplex virus or bacterial infections based on the phosphorylation of radiothymidine derivatives by the viral or bacterial thymidine kinase (TK), which traps them within infected cells [98,99,100]. In a similar manner, ^18^F-labeled fluoromaltotriose, which is selectively taken up by the maltodextrin transporter expressed by most Gram-positive and –negative bacteria has been used to visualize a number of clinically relevant bacterial strains in cell cultures and in living mice [101]. While remdesivir and other nucleoside analogues that suppress SARS-CoV-2 replication are subject to bioactivation and subsequent intracellular trapping, the process is not specific to infected cells and cannot be exploited for radiopharmaceutical development. However, radiolabeling of suitable substrates for RdRP, Mpro or PLpro, which remain to be identified, could produce excellent PET or SPECT tracers to visualize infected cells and would be especially valuable for the development of therapeutic radiopharmaceuticals (see Section 4).

Alternatively, probes for the detection of infected cells could be obtained by radiolabeling of drugs that directly bind to one of the non-structural proteins (i.e., approach 2). Small molecules and peptidomimetics have been recognized as promising candidate vehicles for radionuclide delivery into virus-infected cells [102,103,104], but there is still a lack of studies on the topic, most likely because most antiviral drugs in clinical use are nucleoside analogs and also taken up into uninfected cells. However, as described in Section 2, there are several novel or repurposed non-nucleoside drugs that target SARS-CoV-2 replication, and identification of candidates with little affinity for host molecules could provide the opportunity to develop suitable virus-specific probes.

Finally, the targeting of structural proteins (i.e., approach 3) is exemplified by the use of ^64^Cu-labeled antibodies against Gp120, an SIV envelope protein expressed on the surface of the SI virus itself and of infected cells, for PET-based detection of SIV-infected tissues and free virus in non-human primates [105]. Since SARS-CoV-2 structural proteins appear to be present within but not on the surface of infected cells, radiolabeled analogs of, e.g., the antibodies shown in Table 1 would be expected to selectively target the free virus. On the other hand, small molecules such as arbidol might also cross the cell membrane and bind to newly synthesized structural proteins in cytosol, ER and/or Golgi (Figure 11B3), so that they could possibly be used for simultaneous imaging of the virus and virus-infected cells.

In practice, the development and application of SARS-CoV-2-specific probes could be hampered by the necessary safety precautions and the requirement for imaging systems equipped with special containment measures. On the other hand, virus-specific tracers for longitudinal studies in a single subject can be of immense value for infectious disease research and drug development, which still rely heavily on clinical assays and necropsy of infected subjects at various time points. Considering the continuing threat by emerging coronaviruses and other pathogens, it seems likely that nuclear imaging will become a more important part of antiviral drug development, with an associated increase in the availability of suitably contained imaging hardware. Moreover, as described in the preceding section, initial screening by in vivo studies on PK/ADME properties, toxicity and drug affinity for non-viral targets could also be performed with cultured cells and healthy animals. Likewise, binding to the viral target proteins could initially be assessed using suitable cell-free bead-based assays [106,107], pseudovirions expressing the respective structural proteins [108,109,110] and/or non-infectious replicons expressing the respective non-structural proteins [111,112]. Promising candidates identified might then be transferred to a BSL3/4-approved imaging facility, where they could be used to track the response of infected animals to experimental drugs and/or for studies on SARS-CoV-2 tissue tropism. Alternatively, the main aim of preliminary experiments such as those described above might be the identification of suitable vehicles that could be radiolabeled with therapeutic radionuclides and used to deliver a cytotoxic payload to SARS-CoV-2 virions or infected cells, an approach that will be discussed in the following section.

## 4. Use of Radiopharmaceuticals for the Treatment of COVID-19

The cytotoxic effect of energy deposited by ionizing radiation is the basis for radiation therapeutic methods, which use an external beam source for curative, adjuvant or palliative treatments of cancers not amenable to surgery. During the first half of the 20th century, low-dose radiotherapy (LDRT) was also used as an anti-inflammatory treatment in patients with viral pneumonia [113,114], and similar protocols are still in use for the treatment of acute or chronic inflammatory disorders [115,116]. More recently, a number of small, early-phase studies have been initiated to explore the use of LDRT in patients with COVID-19 pneumonia [117,118,119]. While this approach, which aims to reduce the life-threatening inflammation rather than targeting the virus itself, is interesting, its timing, effectiveness and potential side effects remain controversial [120,121]. Targeted radionuclide therapy (TRT) or endoradiotherapy is an alternative therapeutic strategy that has become a mainstay for the treatment of cancer [122,123]. TRT involves direct administration of a radiolabeled vector for target-specific delivery of a cytotoxic level of radiation to the diseased tissue (Figure 10 bottom). Agents for molecular radiotherapy typically consist of a small molecule, peptide or antibody that serves as the targeting vector and is attached to a macrocyclic or acylic chelator for radiometal complexation [124,125,126]. A major advantage of such designs is that the probe can often be used as a theranostic and radiolabeled with either diagnostic or therapeutic radionuclides, allowing for molecular imaging and TRT to be performed using the same compounds (Figure 11C) [59,127]. Moreover, because the decay of most therapeutic radionuclides is associated with the emission of γ-rays, SPECT can often be used to directly visualize their distribution in vivo [59,123] (reviewed in [128]). With regard to their particle emission, therapeutic radionuclides can be separated into three distinct classes, all of which mainly act by a combination of direct (via ionization) and indirect (via free radical formation) radiation-induced damage to the DNA of target cells (Figure 10 bottom). The most widely used therapeutic radionuclides are α- and β^−^-particle emitters, which decay under emission of a ^4^He^2+^ particle (α-particle) or high energy electron (β^−^-particle) from the isotope’s nucleus [129]. β^−^-Particles have a lower linear energy transfer (LET~0.2 keV/µm) but path lengths of up to several mm in soft tissues, so that they deposit their energy into the nucleus of many (10–1000) cells and are mainly used for the treatment of solid tumors (Figure 10 bottom). α-Particles have a much higher LET (~100 keV/µm) and path lengths of up to a few typical cell diameters (50–100 µm), making them most useful for the treatment of small or disseminated tumors (Figure 10 bottom). The energetic difference between these particles is best illustrated by in vitro studies showing that traversal of the nucleus by one to four α-particles is sufficient to kill a mammalian cell [130,131,132], whereas thousands of β^−^-particles are required for the same effect [133]. A third class of therapeutic radionuclides is made up of Auger electron emitters, which decay by electron capture or internal conversion with subsequent emission of a cascade of low-energy Auger and conversion electrons from the electron shell [129,134]. They deposit their energy over extremely small distances (2–500 nm), which results in relatively high LETs (4–26 keV/µm) but also means that these radionuclides are only effective when emitted in close proximity to sensitive targets such as DNA or the cell membrane (Figure 10 bottom) [134]. Although Auger electron emitters have multiple advantageous characteristics such as a low toxicity when emitted outside of the nucleus, the general requirement for nuclear targeting has also hampered their widespread application for the treatment of cancers [135].

Given the increasing prevalence of highly resistant microorganisms, TRT has been proposed as a potential breakthrough therapeutic approach for the treatment of bacterial, viral and fungal infections [136,137,138,139,140,141]. In contrast to the situation in cancer treatment, where most tumor-associated targets are also expressed in normal tissues, TRT of viral infections could target virus-encoded molecules without closely related homologues in humans, promising a much higher therapeutic index. This form of treatment should consequently allow for selective eradication of virus particles (but see below) or virus-infected cells with little or no damage to neighboring host cells and in a manner that is neither affected by the immunological status of the host nor by drug resistance to conventional treatments. By targeting a highly conserved molecule or domain, it might also be possible to develop broad-spectrum pan-radiopharmaceuticals against a whole class of viruses while minimizing the likelihood that the therapy becomes ineffective when the viruses mutate.

### 4.1. Radionuclide Therapy Targeting SARS-CoV-2 Virions

Analogous to virus-specific nuclear imaging, it has been proposed that COVID-19 could be treated by targeting structural proteins present on the surface of SARS-CoV-2 (Figure 11B3) with radiolabeled antibodies (radioimmunotherapy, RIT) [107]. Considering the small size of SARS-CoV-2 virions (80–120 nm) and the aim to minimize damage to host cells, the most suitable radioactive emitters for this approach would be Auger electron-emitting radionuclides. Due to the relatively low toxicity of these radionuclides when emitted in the cytoplasm or outside of the cell, these radionuclides would need to reach the nucleus to effectively damage the DNA of host cells [142,143,144,145,146,147,148] (Figure 10 bottom). At the same time, Auger electrons with a LET between 0.5 and 10 keV/µm delivered to or into the viral membrane envelope have been estimated to be sufficiently energetic for antiviral effects [107]. In a previous proof-of-concept study, the non-neutralizing monoclonal antibody CR3022, which binds to the SARS-CoV-2 S protein (K_D_ = 6.3 nM) but is cross-reactive and conserved across several coronaviruses [37,38], was radiolabeled with ^131^I and shown to retain its binding properties using cell-free bead-based in vitro assays [107]. A potential drawback of antibody-based radionuclide delivery is that non-neutralizing antibodies as well as neutralizing antibodies at sub-neutralizing levels can result in antibody-dependent enhancement (ADE), a poorly understood phenomenon that is associated with increased infection severity [149,150]. Use of small molecules or peptide analogs targeting SARS-CoV-2 structural proteins as the vehicle might circumvent such effects and, due to their faster clearance from circulation, also reduce the risk of hematological side effects. Even more importantly, these vehicles might exert additional antiviral effects by crossing the cell membrane and binding to the structural proteins produced in infected cells (Figure 11B3). Considering that viral titers in patients infected with SARS-CoV-2 can be very high, it seems unlikely that radiopharmaceuticals targeting the free virus only could effectively reduce the viral load, so that in practice, the use of alternative vehicles such as small molecules appears to be a much more feasible approach. Thus, even though very promising in vitro and in vivo results have been obtained in preclinical studies on the RIT of HIV [137,151], the antibodies used for radionuclide delivery in these studies targeted the viral envelope proteins Gp120 and Gp41, which are not only expressed on the virus itself but also on the surface of infected cells (but see next section).

### 4.2. Radionuclide Therapy Targeting SARS-CoV-2-Infected Cells

A more promising approach for the treatment of COVID-19 could be to use therapeutic radiopharmaceuticals that target one or more of the virus-encoded proteins present in infected cells, so as to eliminate the viral factories. Uptake of the Auger electron-emitter [^125^I]iodide into cells infected with a recombinant measles virus expressing the sodium iodide symporter has previously been shown to effectively suppress viral replication in vitro [152]. While ^125^I (t_1/2_ > 60 d) is clearly unsuitable for TRT of COVID-19, these findings indicate that uptake of Auger electron emitters into infected cells might bring them sufficiently close to the viral RNA for antiviral effects. If this could be confirmed in SARS-CoV-2-infected cells or non-infectious SARS-CoV-2 replicons, radiopharmaceuticals labeled with Auger electron-emitting radionuclides might provide a treatment approach with very little potential for radiotoxic side effects. Other therapeutic radionuclides that would be well suited for this approach are α-particle emitters, while the long range of β^−^-particle emitters might raise concerns with regard to collateral damage to non-infected cells due to crossfire effects. However, as already touched upon in the preceding section, antibodies against the structural proteins Gp120 or Gp41 labeled with the α- & β^−^-emitter ^213^Bi or the pure β^−^-emitter ^188^Re (Table 5) have previously been shown to selectively eradicate HIV-infected cells with minimal cytotoxicity to non-infected cells in vitro and in animal models [137,151]. As discussed above, small molecules or peptidomimetics that can cross the cell membrane and bind to structural proteins in intracellular compartments (Figure 11B3) could possibly be used for delivery of radionuclides into SARS-CoV-2-infected cells. However, given the many candidate drugs described in Section 2.5, the more obvious strategy would be to target one of the non-structural proteins (Figure 11B2). Due to the much lower mass dose required for radionuclide delivery as compared to standard drug treatments, even antiviral drugs that failed to meet the safety and side effect profile required for conventional drug development could potentially be used for TRT of COVID-19. Moreover, and in contrast to radiolabeled antibodies, these molecules would be expected to show rapid accumulation in infected cells and clearance from most normal tissues, reducing the radiation exposure of non-infected host cells and allowing for the use of short-lived radionuclides such as ^213^Bi,^211^At or ^212^Bi (Table 5).

Especially the latter aspect is important because most of the longer-lived therapeutic radionuclides such as ^224^Ra (t_1/2_ = 3.6 d), ^223^Ra (t_1/2_ = 11.43 d) or ^225^Ac (t_1/2_ = 10 d) have multiple long-lived α-emitting daughters in their decay chain [153]. As the recoil energy produced during α-emission is much higher than the energy of any chemical bond, their decay is invariably associated with release of the daughter nuclides from the targeting vector, after which they may redistribute into healthy tissues and produce adverse radiotoxic effects [153]. That said, even therapeutic approaches based on short-lived radionuclides would clearly require a battery of feasibility and toxicity studies in order to ensure their effectiveness, exclude toxic side effects and determine the dose-effect correlation. However, since these studies could be performed with a combination of bead- and cell-based assays, non-infectious SARS-CoV-2 replicons and healthy animals, no special containment measures would be required for a first proof of principle. Depending on the results, suitable candidates could then still be delivered to a nearby BSL3/4-approved laboratory for further evaluation of their in vivo effectiveness in infected animals. While it seems unlikely that TRT alone could ever lead to complete elimination of the virus or virus-infected cells, the approach might be combined with conventional antiviral drugs in order to the reduce the viral burden and thus improve the outcome in severely affected patients. Given the urgency to find a cure, the uncertainty of success in developing a SARS-CoV-2 vaccine and the continuing threat by novel pathogens, it seems to be a good time to investigate the feasibility of alternative treatment strategies.

## 5. Conclusions and Future Perspectives

Taken together, we have described several potential applications of nuclear medicine that could help to fight the current as well as future pandemics with respiratory pathogens such as SARS-CoV-2. Given the lack of previous studies on the topic, most of the approaches could only be discussed in theory and their feasibility remains to be scientifically proven, which is especially important with regard to the therapeutic application of radiopharmaceuticals. However, promising results obtained in preclinical studies on the treatment of other viral infections by radionuclide therapy and the fact that at least the proof-of-concept studies could also be performed in laboratories without special containment measures should encourage further evaluation of this approach as a possible therapeutic option. Likewise, even though the limited availability and risk of disease spreading makes it unlikely that PET or SPECT will ever be used for routine assessment of patients with COVID-19 pneumonia, functional imaging will most likely become important for characterizing the long-term impairments observed in an increasing number of recovered patients. Even more importantly, closer integration of these imaging modalities into antiviral drug development could significantly streamline lead identification, optimization and translation to the clinic. In addition, molecular imaging with radiolabeled antiviral drugs could provide critical input for emerging techniques based on machine learning and artificial intelligence algorithms that may combine information from pharmacokinetic and biodistribution studies with clinical data. Considering the continuing threat by emerging coronaviruses and other pathogens as well as the rapid pace of current technological progress, it also seems likely that improved approaches for preclinical imaging of animals infected with contagious pathogens will become available in the near future, so that nuclear medicine techniques could also contribute to a better understanding of disease transmission, progression and eradication.

## Figures and Tables

**Table 1:** Antibodies and small molecules targeting the SARS-CoV-2 S-protein.

Inhibitor	IC_50_ [µM]	CC_50_ [µM]	Class	Target	Ref.
311mab-31B5	0.0338	NA	nAB	S protein (RBD)	[27]
311mab-32D4	0.0698	NA	nAB	S protein (RBD)	[27]
47D11	0.57	NA	nAB	S protein (RBD)	[28]
COVA1-18	NA	NA	nAB	S protein (RBD)	[29]
COVA2-15	NA	NA	nAB	S protein (RBD)	[29]
CR3022	-	NA	nnAB	S protein (RBD)	[37,38]
Arbidol	4.11 µM	31.79 µM	SM	S protein (S2)	[30]

Abbreviations: NA—not available; IC_50_—concentration for half-maximal inhibition of cell entry; CC_50_—concentration for half-maximal cellular cytotoxicity; nAB—neutralizing antibody; nnAB—non-neutralizing antibody; SM—small molecule.

**Table 2:** Inhibitors of SARS-CoV-2 Mpro.

Inhibitor	IC_50_ [µM]	EC_50_ [µM]	CC_50_ [µM]	Class	MOA	Ref.
GC-376	0.03	3.14–3.37	>100	PA^1^	C	[43]
Inhibitor 11b	0.04	0.72	NA	PA	C	[22]
Inhibitor 11a	0.05	0.53	NA	PA	C	[22]
alpha-ketoamide 11r	0.18	NA	NA	PAK	C	[44]
Calpain inhibitor XII	0.45	0.49–0.78	>50	PAK	C	[43]
Ebselen	0.67	4.67	NA	SM	C	[23]
alpha-ketoamide 13b	0.67	4–5	NA	PAK	C	[44]
Baicalein	0.94	2.94	>200	SM	NC	[49]
Calpain inhibitor II (ALLM)	0.97	2.07–3.70	>100	PA	C	[43]
Tideglusib	1.55	NA	NA	SM	C	[23]
Carmofur	1.82	24.30	NA	SM	C	[23,46]
alpha-ketoamide 13a	2.39	NA	NA	PAK	C	[44]
MG-115 (Proteasome inhibitor)	3.14	NA	NA	PA	C	[43]
MG-132 (Proteasome inhibitor)	3.90	NA	<1	PA	C	[43]
Boceprevir	4.13	1.31–1.95	>100	PAK	C	[43]
Narlaprevir	4.73	NA	NA	PAK	C	[43]
Baicalin	6.41	27.87	>200	SM	NC	[49]
Calpain inhibitor I (ALLN/MG-101)	8.60	NA	NA	PA	C	[43]
Disulfiram	9.35	NA	NA	SM	C	[23]
Proteasome inhibitor I (PSI)	10.38	NA	NA	PA	C	[43]
Calpeptin	10.69	NA	NA	PA	C	[43]
Simeprevir	13.75	NA	NA	PMC	C	[43]
Shikonin	15.75	NA	NA	SM	NC	[23]
PX-12	21.39	NA	NA	SM	C	[23]
N3	NA	16.77	>100	PAE	C	[23,45]

Abbreviations: NA—not available; MOA—mechanism of action; C—covalent inhibitor; NC—non-covalent inhibitor; IC_50_—concentration for half-maximal enzymatic inhibition; EC_50_—concentration for half-maximal antiviral activity in cells; CC_50_—concentration for half-maximal cellular cytotoxicity; PA—peptidomimetic aldehyde; PAK—peptidomimetic α-ketoamide; PAE—peptidomimetic acrylate; PMC—peptidomimetic macrocyle; SM—small molecule; ^1^ prodrug (bisulfite adduct).

**Table 3:** Inhibitors of SARS-CoV-2 PLpro.

Inhibitor	IC_50_ [µM]	EC_50_ [µM]	CC_50_ [µM]	Class	MOA	Ref.
Ebselen	2.0–2.4	4.67	NA	SM	C+ZE	[23,47,48]
GRL-0617	2.4	27.6	NA	SM	NC	[24,52]
Compound 6	5.0	21.0	NA	SM	NC	[52]
Disulfiram	7.5	NA	NA	SM	C+ZE	[48]
7724772	23.5	NA	NA	SM	NC	[52]
6577871	100.7	NA	NA	SM	NC	[52]
9247873	>200	NA	NA	SM	NC	[52]
VIR251	NA	NA	NA	PVME	C	[53]
VIR250	NA	NA	NA	PVME	C	[53]

Abbreviations: NA—not available; MOA—mechanism of action; C—covalent inhibitor; NC—non-covalent inhibitor; ZE—Zn^2+^ ejector; IC_50_—concentration for half-maximal enzymatic inhibition; EC_50_—concentration for half-maximal antiviral activity in cells; CC_50_—concentration for half-maximal cellular cytotoxicity; PVME—peptidomimetic vinyl methyl ester; SM—small molecule.

**Table 4:** Radionuclides commonly used for nuclear medicine imaging.

Radionuclide	Half-Life	Daughters	Decay Type (Probability)	Use
^15^O	2 min	^15^N (stable)	β^+^ (99.9%), EC (0.1%)	PET
^13^N	10 min	^13^C (stable)	β^+^ (99.8%), EC (0.2%)	PET
^11^C	20 min	^11^B (stable)	β^+^ (99.7%), EC (0.3%)	PET
^68^Ga	67 min	^68^Zn (stable)	β^+^ (88.9%), EC (11.1%)	PET
^18^F	110 min	^18^O (stable)	β^+^ (97.0%), EC (3.0%)	PET
^64^Cu	12.7 h	^64^Ni/^64^Zn (stable)	β^+^ (17.9%), EC (43.1%), β^-^ (39.0%)	PET
^99m^Tc	6 h	^99^Tc ^a^	[γ-Ray (88%), IC (12%)]	SPECT
^123^I	13.2 h	^123^Te ^b^	EC (100%) [γ-Ray (84%), IC (16%)]	SPECT
^111^In	67 h	^111^Cd (stable)	EC (100%) [γ-Ray (100%)]	SPECT

Abbreviations: EC-electron capture; IC-internal conversion; ^a^ half-life > 200k years; ^b^ half-life > 3.2 × 10^16^ years.

**Table 5:** Radionuclides with potential for anti-viral treatment.

			α -Particle Emission			β -Particle Emission			Auger Emission		
Radio-			P^a^	E_max_	Range	P^a^	E_max_	Range	P^a^	E_max_	Range
Nuclide	Daughters	Half-Life	[%]	[MeV]	[µm]	[%]	[MeV]	[mm]	[%]	[keV]	[µm]
^213^Bi		46 min	2.2	5.9	50	97.8	0.5	1.7	-	-	-
	^213^ Po→^209^Pb	4.2 µs	100	8.4	90	-	-	-	-	-	-
	^ 209 ^ TI→^209^Pb	2.2 min	-	-	-	100	0.6	2.3	-	-	-
	^ 209 ^ Pb→^209^Bi (stable)	3.3 h	-	-	-	100	0.2	0.5	-	-	-
^212^Bi		1 h	35.9	6.1	50	64.1	0.8	3.3	-	-	-
	^ 212 ^ Po→^208^Pb (stable)	299 ns	100	8.8	100	-	-	-	-	-	-
	^ 208 ^ TI→^208^Pb (stable)	3.1 min	-			100	0.3–0.6	0.9–2.3	-	-	-
^211^At		7.2 h	41.8	5.9	50	-	-	-	58.2	93	<0.5
	^ 211 ^ Po→^207^Pb (stable)	516 ms	100	7.4	70	-	-	-	-	-	-
	^ 207 ^ Bi→^207^Pb (stable)	33 yrs^b^	-	-	-	-	-	-	100	88	<0.5
^188^Re	^188^Os (stable)	17.0 h	-	-	-	100	2.0	10	-	-	-
^166^Ho	^166^Er (stable)	28.8 h	-	-	-	100	1.8	9	-	-	-
^103m^Rh	^103^Rh (stable)	56 min	-	-	-	-	-	-	100	39.7	<0.5
^161^Ho	^161^Dy (stable)	2.48 h	-	-	-	-	-	-	100	NA	<0.5
^123^I	^123^Te (stable)	13.2 h	-	-	-	-	-	-	100	32	<0.5
^111^In	^111^Cd (stable)	67 h	-	-	-	-	-	-	100	26	<0.5

^a^ Probability; ^b^ Despite its long half-life, ^207^Bi will not contribute significantly to the radiation dose during radionuclide therapy [135]. Note that range refers to maximum range in soft tissues.

**Figure A1:**
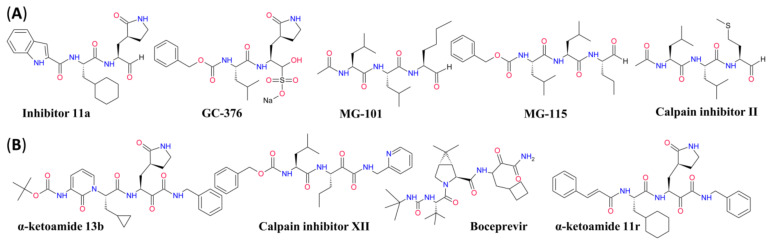
Structure of selected peptidomimetic SARS-CoV-2 Mpro inhibitors with electrophilic aldehyde (**A**) or α-ketoamide (**B**) warhead. For mechanism of action and pharmacological properties see Figure 5 and Table 2 in the main text.
